# Folk beliefs about genetic variation predict avoidance of biracial individuals

**DOI:** 10.3389/fpsyg.2015.00357

**Published:** 2015-04-07

**Authors:** Sonia K. Kang, Jason E. Plaks, Jessica D. Remedios

**Affiliations:** ^1^Institute for Management and Innovation, University of Toronto MississaugaMississauga, ON, Canada; ^2^Rotman School of Management, University of TorontoToronto, ON, Canada; ^3^Department of Psychology, University of TorontoToronto, ON, Canada; ^4^Department of Psychology, Tufts UniversityMedford, MA, USA

**Keywords:** automatic/implicit processes, categorization, lay theories, person perception, social neuroscience

## Abstract

People give widely varying estimates for the amount of genetic overlap that exists between humans. While some laypeople believe that humans are highly genetically similar to one another, others believe that humans share very little genetic overlap. These studies examine how beliefs about genetic overlap affect neural and evaluative reactions to racially-ambiguous and biracial targets. In Study 1, we found that lower genetic overlap estimates predicted a stronger neural avoidance response to biracial compared to monoracial targets. In Study 2, we found that lower genetic overlap estimates predicted longer response times to classify biracial (vs. monoracial) faces into racial categories. In Study 3, we manipulated genetic overlap beliefs and found that participants in the low overlap condition explicitly rated biracial targets more negatively than those in the high overlap condition. Taken together, these data suggest that genetic overlap beliefs influence perceivers’ processing fluency and evaluation of biracial and racially-ambiguous individuals.

## Introduction

In general, perceivers view racial categories as *natural kinds* whose members share a deep, underlying *essence* that governs a range of visible (e.g., skin tone) and invisible (e.g., intelligence) traits (Medin and Ortony, 1989; Yzerbyt et al., 2001; Haslam and Whelan, 2008). An essentialist view of race is associated with the belief that racial categories are good predictors of personality and behavior (Rothbart and Taylor, 1992; Yzerbyt et al., 2001). As such, essentialist beliefs have been found to predict racial stereotyping and prejudice (McGarty et al., 2002; Keller, 2005; Bastian and Haslam, 2006; Condit, 2008; Williams and Eberhardt, 2008; Chao et al., 2013).

Surprisingly little research, however, has investigated how exactly people mentally represent the biological roots of essence. What—according to laypeople—creates a racial essence? A likely candidate is genes. In the present studies, we examined people’s underlying assumptions about the relationship between genetic variation and race.

Recent evidence suggests that people generally consider between-group differences that are ascribable to genetic factors to be more significant and immutable than differences rooted in social or environmental factors (Dar-Nimrod and Heine, 2006; Schnittker, 2008; Williams and Eberhardt, 2008; Dar-Nimrod and Heine, 2011). But what precisely do people believe about variation in the human genome within and between common race categories like “Black,” “White,” or “Asian”? How much individual variation is there in such beliefs? And how, if at all, might variation in beliefs about human genetic overlap affect perceivers’ evaluations of biracial people, who straddle the boundaries between categories?

While evidence from the Human Genome Project suggests that humans, regardless of race, share 99.9% of their genetic material (Feldman et al., 2003; Ossorio and Duster, 2005), recent studies demonstrate that laypeople give widely varying estimates of the percentage of genetic material that humans share (with a mean of ∼68%; Plaks et al., 2012). These varying estimates have behavioral consequences. For example, Plaks et al. (2012) demonstrated that the lower the estimate of intergroup genetic overlap, the greater the tendency to visually perceive discrete (“either/or”) boundaries between races. In other words, beliefs guided perception: a stronger *a priori* belief in discrete (genetically-based) categories predicted a stronger tendency to perceive a continuously-varying array of morphed faces as conforming to two discrete categories.

In the current work, we extend these findings by focussing specifically on how people react to faces in the middle of this continuous array—the blended, biracial faces that are most racially-ambiguous. Here, we aim to gain a more specific understanding of (a) whether beliefs about genetic overlap influence perceivers’ evaluations of ambiguous faces, and (b) what mechanisms may begin to explain any differences in evaluation. We do so by using a neural measure of avoidance (Study 1), a behavioral measure of processing disfluency (Study 2), and an explicit measure of trait judgments (Study 3).

### The Case of Biracial Target Persons

We theorized that biracial targets pose a unique perceptual and conceptual challenge because they are more likely than others to be racially-ambiguous, making them difficult to categorize discretely (Remedios and Chasteen, 2013). We hypothesized that this processing disfluency when perceiving biracial individuals would be especially pronounced for those with a low genetic overlap perspective. “Low overlap” perceivers expect different people to be relatively genetically distinct, with a clear threshold where one person or group ends and another begins (Plaks et al., 2012). The either/or nature of this threshold generally encourages assimilation of faces into one category or the other (Eberhardt et al., 2003; Sanchez et al., 2011). We expected, however, that racially-ambiguous faces would resist easy classification, challenging the assumption of discrete categories. Therefore, we hypothesized that, compared to individuals who believe in high genetic overlap, individuals with lower overlap beliefs would experience greater difficulty classifying biracial faces, exhibit higher levels of neural avoidance toward such faces, and explicitly evaluate them more negatively. In contrast, because perceivers with higher overlap beliefs consider racial categories to be comparatively indistinct, they should experience less confusion when they encounter ambiguous faces. Thus, these participants should be less motivated to avoid or denigrate ambiguous targets.

Our approach extends previous studies that have investigated how beliefs about race influence judgments of mono- and biracial targets. For example, Blascovich et al. (1997) found that participants who were high (vs. low) in anti-Black prejudice took longer to categorize racially-ambiguous faces. We posit that genetic overlap beliefs are conceptually and empirically distinct from anti-Black prejudice (see Plaks et al., 2012). Genetic overlap beliefs are descriptive beliefs about all humans, rather than evaluative stances toward any specific group. Thus, a person may sincerely espouse egalitarian ideals, but believe in low genetic overlap—a position captured by the phrase “separate but equal” (see Park and Judd, 2005).

Other researchers have focussed more specifically on the effects of perceivers’ *a priori* beliefs on the perception of mono- and biracial targets. For example, Eberhardt et al. (2003) reported that participants with an “entity” theory (the belief that traits are fixed) were more likely than those with an “incremental” theory (the belief that traits are malleable) to categorize biracial faces as either Black or White. The present studies seek to extend that work by uncovering further beliefs about genetics that might, in fact, provide a basis for assumptions about fixedness/malleability.

## Study 1

All studies reported here were approved by the Office of Research Ethics at the University of Toronto.

In Study 1, we examined whether genetic overlap beliefs would influence neural avoidance by using electroencephalography (EEG) to record participants’ neural responses while they viewed films in which faces slowly and continuously morphed from Black to White. We focused on frontal EEG asymmetry, which is thought by many to index the motivation to approach or avoid a given stimulus (Coan and Allen, 2003). Greater relative left frontal activity has been associated with approach motivation; greater relative right frontal activity has been associated with avoidance motivation (Harmon-Jones and Allen, 1997; Davidson, 1998). We hypothesized that if participants with low overlap beliefs found racially-ambiguous targets to be perceptually challenging, they would display a pronounced pattern of greater relative left frontal activity at the beginning and end of the Black–White morph videos (when the faces appear monoracial and racial categorization is easier) but greater relative right frontal activity toward the middle (when racial ambiguity is maximal). On the other hand, we did not expect to see this difference between the ends vs. the middle of the transitions among participants with high overlap beliefs. Specifically, we were interested in differences in neural markers of avoidance when low vs. high overlap perceivers attend to racially-ambiguous faces.

### Method

#### Participants

Participants were 25 (60% female) undergraduate students (44% White, 28% East/South East Asian, 12% Middle Eastern, 8% South Asian, 8% Other).

#### Materials

##### Black-white morph videos

**FIGURE 1:**
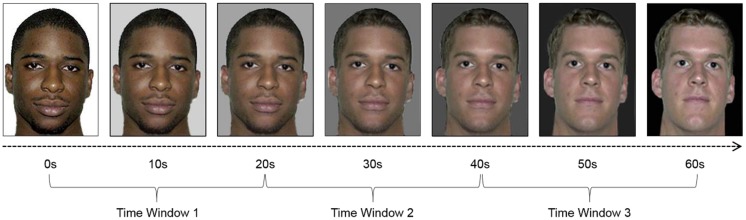
**Sample images taken from one of the 60-s videos of faces morphing from Black to White used in Study 1.** Faces changed from Black at 0 s to White at 60 s; Time windows used for analysis are as indicated. Note that the start and end faces were matched on attractiveness, ear shape, and head shape, size, and position and that overall image luminance was held constant by progressively darkening the background color.

We created a series of videos showing faces changing from one to another using 15 pairs of standardized and normed Black and White faces of undergraduate-age males (Goff et al., 2008). The faces were matched on attractiveness rating, head and ear shape, head size, and head position. Using these pairs, we created fifteen 60-s videos showing faces morphing from a Black face at 0 s to a White face at 60 s. Luminance was held constant by progressively darkening the background (as the face became lighter). Sample images taken from one of these videos are displayed in **Figure 1**. Creating the facial morphs in this way in this and the subsequent studies allows for more precise control over the “mixture” of the biracial faces than is possible with photographs of real biracial individuals. With facial morphs we can precisely control the percentage of facial features drawn from each of the “parent” faces, which is not possible with photographs of real people.

#### Procedure

After providing informed consent, participants were connected to the EEG system. The 15 Black-to-White face morph videos were presented in random order for 60 s each. Participants watched the videos passively, without making any responses. After each video, participants were given the opportunity to take a short break. The video display and EEG recording were synchronized. Given EEG’s high temporal acuity, we could assess, in real time, variation in alpha asymmetry as a function of each face’s racial composition.

Electroencephalography recording was stopped after the video task and participants were given time to wash their hair. After this break, participants completed additional, ostensibly unrelated questionnaires that were said to be provided by other researchers in the department. Embedded in these measures were two questions (order counter-balanced) designed to assess genetic-overlap beliefs:

(1) If you were to choose two people at random from the entire world, what percentage of genetic material would they have in common?
(2) If you were to choose two people at random who happened to be from the same racial background, what percentage of genetic material would they have in common?

Question “1” has been used in previous research to index beliefs about genetic overlap between racial groups (Plaks et al., 2012). This measure has been demonstrated to be empirically distinct from such potential confounds as political orientation and Need for Cognition (Plaks et al., 2012)^1^.

The purpose of Question “2” (about genetic material shared *within* racial groups) was to put participants’ responses to Question “1” into context, allowing us to gain additional detail regarding how assumptions about overall genetic variation relate to assumptions about within-group variation in particular. Are these estimates positively correlated, negatively correlated, or unrelated? In other words, do people believe that high between-group difference implies low within-group difference (consistent with research on group homogeneity effects (e.g., Judd et al., 1991) or do they believe that high between-group difference also implies high within-group difference (consistent with a general perspective that treats each individual as a distinct biological entity)?

Next, participants completed a short demographics questionnaire which included a question about political orientation (“In general, what is your position on political, social, and economic issues?”; 1 = very liberal…6 = very conservative). We included this item in light of recent findings indicating that higher conservatism predicts a stronger tendency to perceive biracial faces as Black (Krosch et al., 2013). Finally, participants were fully debriefed before leaving the lab.

### Results

#### Genetic Overlap Beliefs

As observed in previous research, participants’ responses to the genetic overlap questions varied widely: Question “1” (general overlap estimate; *M* = 71.34%, SD = 32.90%, range: 1–100%); Question “2” (within-group overlap estimate; *M* = 84.10%, SD = 18.87%, range: 40–100%). The correlation between the two items was positive and high, *r* = 0.91, *p* < 0.001.

#### EEG Recording and Calculating Frontal Asymmetry

Electroencephalography was recorded from 32 Ag/AgCl electrodes in a stretch-lycra cap. Signals were amplified (60 Hz notch filter) and digitized at 560 Hz using ASA acquisition hardware (Advanced Neuro Technology) with average-ear references and forehead ground. Impedances were kept below 5 kΩ and frequencies below 1 Hz and above 100 Hz were digitally filtered (96 dB, zero-phase shift). Movement artifacts were detected with a -75 and +75 μV threshold. Two second epochs were extracted through a Hamming window (75% overlap). To examine alpha asymmetry we used a fast Fourier transform to extract power within the alpha band (8.00–13.00 Hz). We created three time windows (0–20 s, “Black”; 20–40 s, “Biracial”; 40–60 s, “White”) and calculated the relative left vs. right frontal activation asymmetry as the log of the alpha power at electrode site F4 (right) minus the log of the alpha power at electrode site F3 (left; Harmon-Jones and Allen, 1997). Higher (more positive) scores indicate greater relative left (approach) compared to right (avoidance) activity (alpha power and cortical activity are inversely related).

In order to examine genetic overlap beliefs continuously, we followed the recommendations of Van Breukelen and Van Dijk (2007) and submitted the frontal asymmetry values to a repeated-measures ANCOVA with time window [time 1 (Black): 0–20 s; time 2 (Biracial): 20–40 s; time 3 (White): 40–60 s] entered as a within-subjects variable and responses to the two genetic overlap questions (continuous, centered) entered as covariates (see also Plaks et al., 2012). Time windows are displayed in **Figure 1**. The overall mean frontal asymmetry values for each time period were as follows: time 1 (Black): *M* = 0.0501, SD = 0.0853; time 2 (Biracial): *M* = -0.00767, SD = 0.137; time 3 (White): *M* = 0.0458, SD = 0.130.

This analysis revealed a significant main effect of time window, *F*(2,44) = 6.01, *p* < 0.01, $\eta_{p}^{2}$ = 0.21, and significant time × estimate interactions for Question “1” (general overlap estimate), *F*(2,44) = 3.40, *p* < 0.05, $\eta_{p}^{2}$ = 0.13, and Question “2” (within-group overlap estimate), *F*(2,44) = 6.61, *p* < 0.01, $\eta_{p}^{2}$ = 0.23. Within-subjects contrasts revealed a significant quadratic trend for both interactions: Question “1”: *F*(1,22) = 6.37, *p* < 0.05, $\eta_{p}^{2}$ = 0.22; Question “2”: *F*(1,22) = 12.44, *p* < 0.01, $\eta_{p}^{2}$ = 0.36^2^.

**FIGURE 2:**
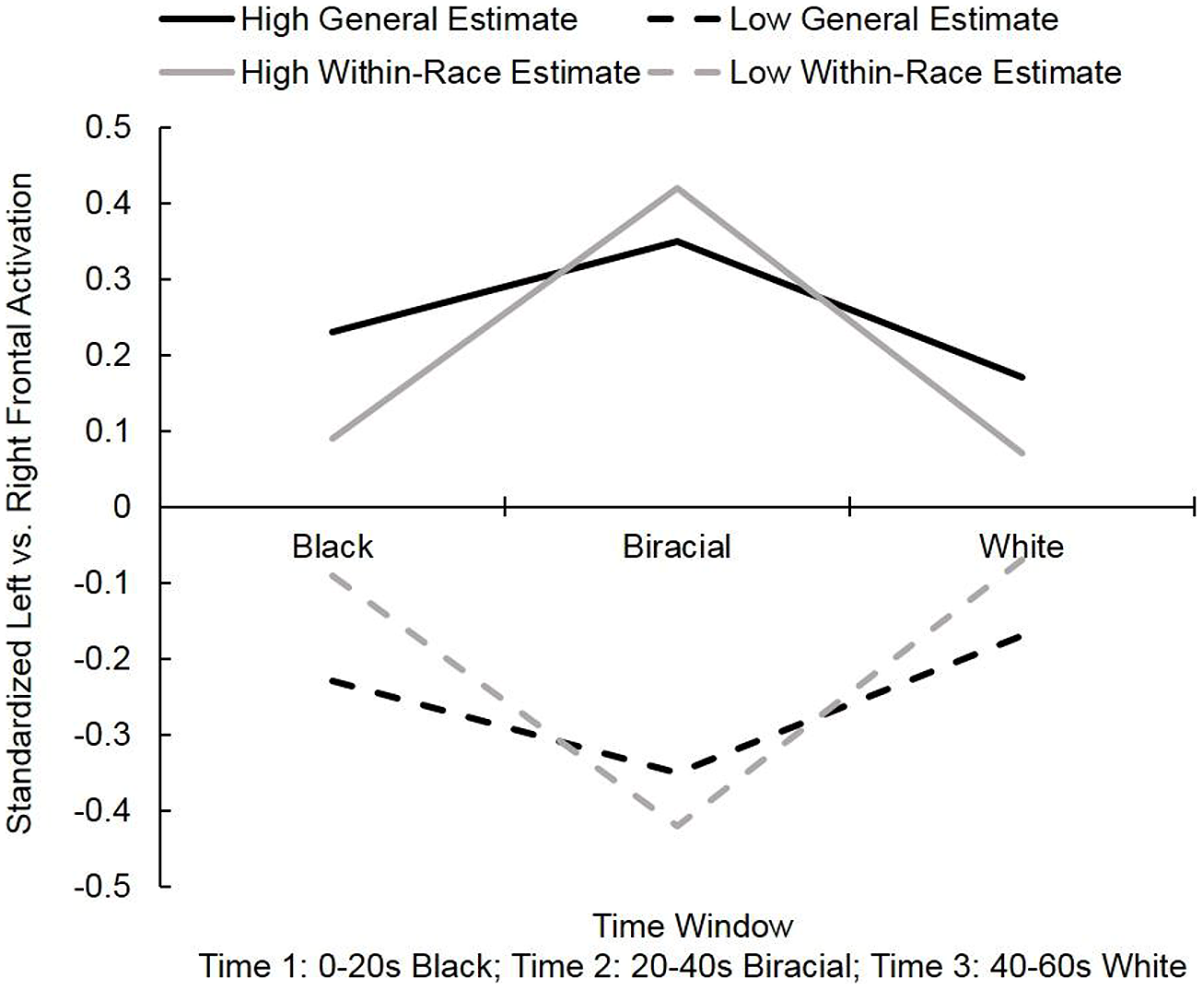
**Mean standardized left vs. right frontal activation observed while participants viewed 60-s videos of faces morphing from Black to White [(*y* > 0 = greater relative left activation (approach); *y* < 0 = greater relative right activation (withdrawal)] in Study 1.** “High” and “low” values represent the frontal asymmetry values of participants at 1 SD above and below the mean on the general and within-race genetic overlap questions.

To probe these interactions, we regressed the frontal asymmetry values at each time window onto the genetic overlap estimates. The results for participants at 1 SD above and below the mean on the genetic overlap questions are displayed in **Figure 2**. First, we examined estimates of general genetic overlap (Question “1”). At time 2, when the faces were most racially-ambiguous, lower estimates of genetic overlap between two randomly-selected people marginally predicted greater relative *right* frontal activation (avoidance), β = 0.35, *t*(23) = 1.80, *p* = 0.09. At time 1 [β = 0.23, *t*(23) = 1.15] and time 3 [β = 0.17, *t*(23) = 0.84], estimates of genetic overlap did not significantly predict frontal asymmetry values, *p*s > 0.25. Thus, as predicted, a general assumption of clear, genetically-based distinctions between two randomly-drawn humans elicits a comparatively negative response toward people who are not readily classifiable.

Next, we examined estimates of within-race genetic overlap (Question “2”). Once again, at time 2, lower estimates of overlap predicted more relative right frontal activation (avoidance), β = 0.42, *t*(23) = 2.23, *p* < 0.05. Estimates of genetic overlap within racial groups did not significantly predict frontal asymmetry values at time 1 [*β* = 0.09, *t*(23) = 0.44] or time 3 [*β* = 0.07, *t*(23) = 0.32], *p*s > 0.65. This pattern suggests that people who give lower within-group overlap estimates expect individuals to differ visibly from one another – even when those individuals are from the same racial group. Visual evidence of a continuous blur from one individual to another violates the expectation of clear boundaries between individuals and thus elicits a relative avoidance response. Note that this neural avoidance pattern was no longer significant once the target face clearly belonged to another person (at time 3).

*A priori*, it might have been conceivable that high within-group estimates would predict lower “whole world” estimates (i.e., more within-group sameness implies greater between-group difference). In these data, however, the two estimates were highly, *positively* correlated. Moreover, both estimates predicted EEG responding in a similar fashion. Thus, a general representation of low genetic overlap mattered more than the within- vs. between-group distinction.

We hypothesize that a primary reason why people with low genetic overlap beliefs are inclined to avoid biracial faces is because such faces disrupt processing fluency—they are harder to classify. Study 1, however, contained no measures of processing fluency. Thus, in Study 2, we used a face-classification task to examine whether belief in lower genetic overlap would predict more difficulty in categorizing biracial faces compared to monoracial faces.

## Study 2

### Method

#### Participants and Design

Participants were 449 (47% female, mean age = 48.70, SD* =*13.91) residents of the United States recruited via Qualtrics Panels in exchange for $4.20 compensation (equivalent to ∼$25/h; 59% White, 5% African American, 4% Latino, each remaining category including biracial <3%). Participants were randomly assigned to one of two classification conditions (described below) and all participants saw two types of faces, resulting in a 2 (classification condition: race, control) × 2 (face type: monoracial, biracial) mixed design.

#### Materials and Procedure

After providing informed consent, participants completed seven questions (randomized) to assess beliefs about genetics. Three questions asked for estimates of genetic variation between two humans selected from: (1) the entire world, (2) the same race, and (3) different races. Four final multiple-choice questions were included as a quiz of knowledge of genetics (e.g., “The human genome is made up of how many pairs of chromosomes?” “An organism’s physical appearance or observable characteristics is known as its_____.”).

To obfuscate the connection between the genetics questions and the face classification task, participants also completed an anagram task. Participants were asked to rearrange the letters in a string of text (e.g., AEMDR) to produce a word (e.g., DREAM), and were given 120 s to complete as many items as possible.

**FIGURE 3:**
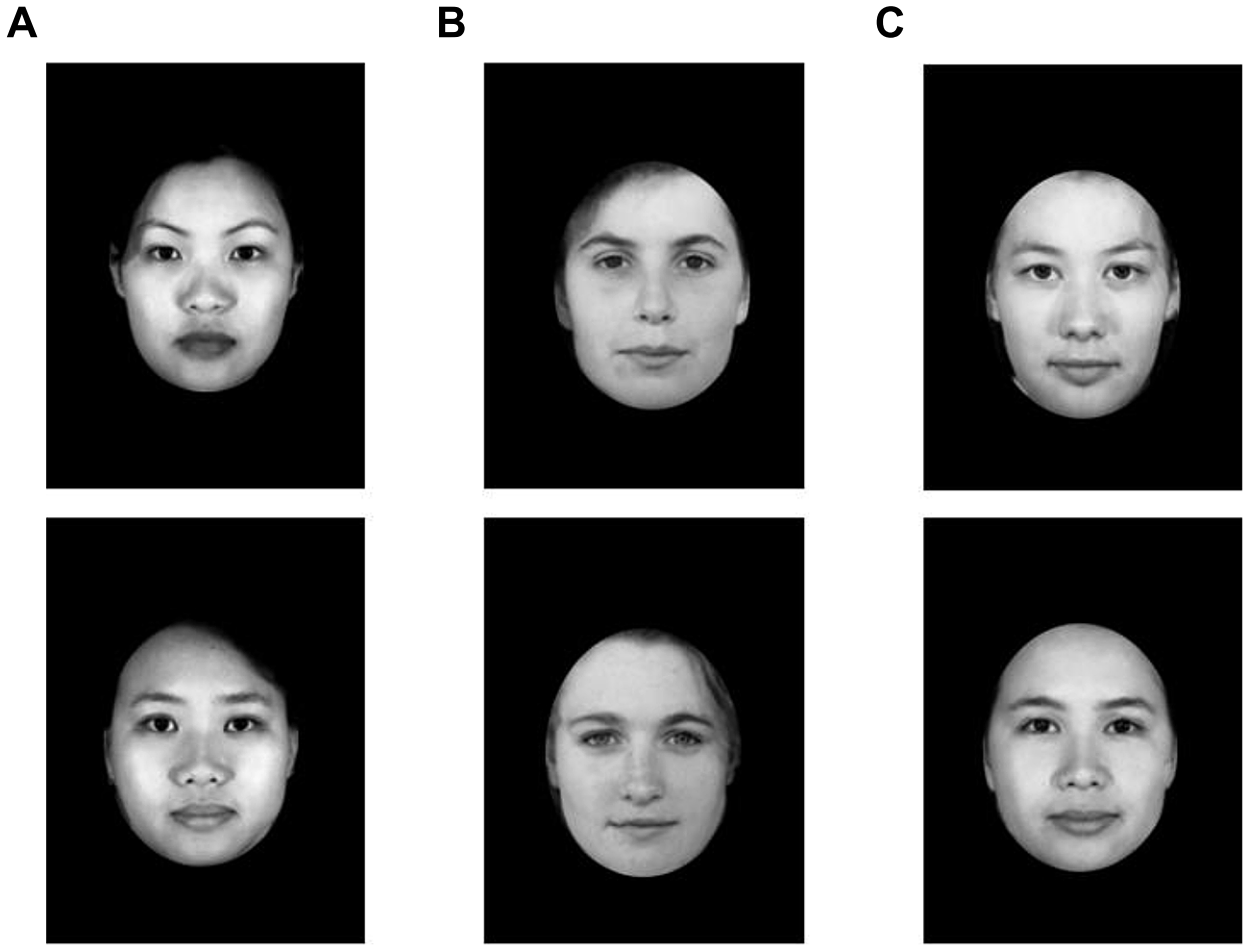
**Sample photographs from Study 2.** Stimuli used in this study included 24 monoracial faces (12 Chinese morphs and 12 Caucasian morphs), and 24 biracial faces (Chinese-Caucasian morphs). Column **(A)**: monoracial Chinese; column **(B)**: monoracial Caucasian; column **(C)** biracial Chinese-Caucasian.

Participants also completed the classification task reported by Halberstadt and Winkielman (2014; Study 3). Participants viewed a series of 48 stimulus faces which were presented in the center of the screen for 3000 ms, following a 500 ms fixation cross. Faces were created using 24 photographs of Chinese individuals and 24 photographs of Caucasian individuals from the University of Western Australia’s “Facelab” database (see Halberstadt and Winkielman, 2014). Morphing software was used to blend each photograph with one same-race photograph and one other-race photograph to create 48 stimulus photographs: 24 monoracial faces (12 Chinese morphs and 12 Caucasian morphs), and 24 biracial faces (the Chinese-Caucasian morphs). In other words, both the monoracial stimuli and biracial stimuli were morphs created from the faces of two different people. This was done to rule out the potential confound that there are inherent differences (e.g., perceived realism) between morphed faces and the faces of real people, independent of racial composition. Sample photographs used in this study are displayed in **Figure 3**.

Participants in the race classification condition pressed the “A” key if the face was Caucasian or the “L” key if the face was Asian. Those in the control condition pressed the “A” key if the person was “feeling positive” or the “L” key if the person was “feeling negative” at the time the photograph was taken. The links between keyboard letters and response options were counter-balanced between participants. The inclusion of an emotion classification condition follows Halberstadt and Winkielman (2014), who found that people were slower to classify the biracial faces only in the race classification condition.

Faces were presented with an inter-trial interval of 5000 ms. If reaction times (RTs) exceeded 3000 ms, the program prompted participants to respond more quickly. Immediately after each classification, participants were asked to rate the attractiveness of the face just presented (1 = “not attractive”…9 = “very attractive”). Finally, participants completed a short demographics survey and were fully debriefed.

The overall order of tasks was varied such that the genetics questions occurred before or after the classification task, and these two tasks were always separated by the anagrams task. The order of the genetics questions and classification task were randomized in this way to control for the possibility that responses on one task might influence responses on the next task, despite the interposed anagrams task.

### Results

#### Genetic Overlap Beliefs

As observed in Study 1, participants’ responses to the human genetic overlap questions varied widely: Question “1” (general overlap estimate; *M* = 51.28%, SD = 35.78%, range: 0–100%); Question “2” (within-group overlap estimate; *M* = 65.94%, SD = 30.55%, range: 0–100%); Question “3” (between-group overlap estimate; *M* = 53.00, SD = 34.34, range: 0–100%). The correlations among the three items were positive and high, all *r*s > 0.74, all *p*s < 0.001.

#### Reaction Times

Participants with RTs greater than two SDs from the mean were removed (0.8%). All remaining RTs were log transformed. Additional participants’ data were removed due to failure to complete primary dependent measure items (*n* = 12) or incoherent responding (*n* = 8), resulting in a final total of 393 participants.

We examined the effects of genetic overlap beliefs on RTs to categorize biracial faces compared to monoracial faces with a repeated-measures full factorial ANCOVA. Face type (monoracial vs. biracial) was entered as a within-subjects variable, classification condition [race classification vs. emotional (control) classification] was entered as a between-subjects variable, and responses to the two genetic overlap questions used in Study 1 (“whole world” and “same race”) were entered as covariates (continuous, centered)^3^^,^^4^ . This analysis revealed main effects for the following: (1) face type, *F*(1,386) = 16.20, *p <*0 .001, $\eta_{p}^{2}$ = 0.04 (indicating that, overall, participants responded slower to biracial targets, *M* = 1602.35 ms, SD = 434.74 ms, than to monoracial targets, *M* = 1500.01 ms, SD = 421.37 ms; (2) Question “1” (general overlap estimate), *F*(1,386) = 8.19, *p* < 0.01, $\eta_{p}^{2}$ = 0.02; and (3) Question “2” (within-group overlap estimate), *F*(1,386) = 5.50, *p* < 0.02, $\eta_{p}^{2}$ = 0.01. These latter two effects indicated that higher estimates of genetic overlap generally predicted slower RTs.

In addition, the following two-way interactions emerged: (1) face type × classification condition, *F*(1,386) = 4.99, *p* < 0.05, $\eta_{p}^{2}$ = 0.01, replicating ((s))Halberstadt and Winkielman (2014) finding that it took longer to classify biracial faces according to race; (2) face type × “whole world” general overlap estimate (Question “1”), *F*(1,386) = 11.83, *p* < 0.001, $\eta_{p}^{2}$ = 0.03, and (3) face type × “same race” within-group estimate (Question “2”), *F*(1,386) = 7.65, *p* < 0.01, $\eta_{p}^{2}$ = 0.02. These latter two interactions indicated that the tendency to respond slower to biracial (vs. monoracial) faces varied according to one’s belief in genetic overlap: the lower one’s genetic overlap estimates, the longer it took to classify biracial faces.

The analysis also revealed the predicted three-way interactions for face type × classification condition × “whole world” general overlap estimate (Question “1”), *F*(1,386) = 7.28, *p* < 0.01, $\eta_{p}^{2}$ = 0.02 and face type × classification condition × “same race” within-group estimate (Question “2”), *F*(1,386) = 9.28, *p* < 0.01, $\eta_{p}^{2}$ = 0.02. These interactions indicated that the tendency to respond slower to biracial faces as a function of belief in genetic overlap varied according to whether the participant was asked to classify the target’s race or the target’s emotional state.

To probe these interactions, we conducted analogous ANCOVAs within each classification condition. As expected, when the participants’ task was to classify the target’s emotional state, genetic overlap beliefs did not influence RTs, all *F*s < 3.30, all *ps* > 0.07.

However, when the task was to classify the target’s race, a face type × “whole world” general overlap estimate (Question “1”) interaction emerged, *F*(1,179) = 6.52, *p* = 0.01, $\eta_{p}^{2}$ = 0.04, indicating that RTs to biracial vs. monoracial faces varied as a function of genetic overlap beliefs. More specifically, the lower the estimate of genetic overlap between two humans selected from the whole world, the longer it took participants to classify biracial faces, β = -0.15, *t*(182) = -2.05, *p* < 0.05. Thus, consistent with our hypothesis, belief in lower genetic overlap was associated with experiencing greater difficulty when classifying biracial (but not monoracial) faces according to their race.

In this analysis, the face type × “same race” within-group estimate (Question “2”) interaction was no longer significant, β = -0.08, *t*(182) = -1.09, *p* > 0.25. Thus, when included simultaneously, beliefs about “whole world” genetic overlap appeared to be the better predictor of RTs to biracial vs. monoracial faces. Raw RT means and SDs are presented in **Table 1**.

**Table 1:** Study 2 response times.

	Race classification condition		Emotional classification condition (Control)	
	Monoracial target	Biracial target	Monoracial target	Biracial target
**“Whole World” estimate**				
High overlap	1358.65 (351.52)	1578.27 (330.27)	1633.36 (403.21)	1621.93 (449.68)
Low overlap	1465.35 (340.24)	1826.99 (432.63)	1572.54 (441.79)	1586.97 (422.95)
** “Same Race” estimate**				
High overlap	1421.88 (388.92)	1654.74 (405.36)	1614.96 (442.47)	1584.80 (488.47)
Low overlap	1456.56 (340.01)	1709.14 (430.29)	1553.44 (427.29)	1568.44 (488.47)

*Mean response times (in milliseconds) to categorize monoracial and biracial target persons as a function of genetic overlap beliefs in Study 2. SDs appear in parentheses. “High” vs. “Low” Overlap are reported at 1 SD above and below the sample mean.*

Additional analyses that included the “different race” genetic overlap question as the predictor variable did not yield any significant effects (all *p*s > 0.25). This is most likely because asking participants to explicitly compare the genetics of different racial groups activates a host of social desirability concerns, however, we believe that the “whole world” question may serve as an effective proxy for the “different race” question.

#### Attractiveness Ratings

We conducted analogous analyses examining the effects of genetic overlap beliefs on attractiveness ratings of biracial face morphs compared to monoracial face morphs. Face type (monoracial vs. biracial) was entered as a within-subjects variable, classification condition [race classification vs. emotional (control) classification] was entered as a between-subjects variable, and responses to the “whole world” and “same race” genetic overlap questions were entered as covariates (continuous, centered). This analysis revealed no significant main effects or interactions, all *F*s < 0.35^5^. In other words, although believers in low genetic overlap found biracial faces more difficult to categorize, they did not find them less attractive.

In sum, while we acknowledge that the observed effect sizes are small, Study 2 provides response time evidence that the lower the belief in genetic overlap between two humans, the greater the difficulty in racially classifying biracial (but not monoracial) target faces. This supports the notion that the belief in different people as genetically distinct entities encourages dichotomous (vs. continual) racial classification (Plaks et al., 2012). Study 2 goes on to suggest that when a target person thwarts easy racial classification, believers in low overlap find the task more difficult.

How does this difficulty with racial categorization translate into behavior toward a racially-ambiguous target? Based on Study 1, one answer to this question might be greater avoidance of biracial (vs. monoracial) targets. It is possible, however, that low overlap participants in Study 1 avoided biracial targets not because they evaluated them negatively *per se*, but simply in order to avoid the feeling of confusion when faced with a difficult categorization task. Thus, in Study 3 we examined whether the differences observed in Studies 1 and 2 would translate into explicit negative evaluations of a biracial target. If so, this would suggest that low overlap believers’ response to biracial targets extends beyond their own feelings of confusion (“I don’t know how to categorize this person”) to the actual attribution of negative traits to the target (“he is disagreeable”).

In Study 3, we also sought to expand the methodologies employed in Studies 1 and 2 by manipulating beliefs about shared genetic material. Our attempt to manipulate genetic belief was motivated by two goals. First, if effects similar to those of Study 1 and 2 were found with manipulated beliefs, this would suggest that beliefs about genetic overlap help to cause different patterns of race perception. Second, if those randomly assigned to the high genetic overlap condition were to evaluate biracial individuals less negatively than those in a low genetic overlap condition, this would have practical implications for prejudice reduction programs.

Why might one hypothesize that these beliefs can be manipulated? Most people have little intuitive knowledge about genomic science and few strong convictions about genetic variability. Thus, both the high and low overlap perspectives may seem believable to most people. Put differently, both perspectives may be cognitively available in long-term memory, but either may be made more accessible through persuasive primes (e.g., Bargh et al., 1988). In previous work, researchers have successfully manipulated general implicit theories of human traits and abilities (e.g., Plaks and Chasteen, 2013, Study 3; Plaks et al., 2009, Study 3) as well as specific beliefs about genetic overlap (Plaks et al., 2012, Study 2). Therefore, Study 3 allowed us both to examine the manipulability of genetic overlap beliefs and to investigate their impact on evaluations of racially-ambiguous targets.

## Study 3

### Method

#### Participants

Participants were 171 (48% female) residents of the United States recruited online via Amazon Mechanical Turk in exchange for $0.50 compensation (79% White, 6% African American, 5% East/Southeast Asian, 4% Latino, 6% other categories (including Pacific Islander, Native American, and biracial). The mean age was 32.06 (SD = 10.51). The mean political orientation was 1.23 (SD = 2.62) on a scale from -5 (extremely conservative) to +5 (extremely liberal, a score which corresponded to “slightly liberal.”

#### Materials

#### Genetic Overlap Manipulation

Participants were randomly assigned to one of two genetic overlap belief conditions. In the high overlap condition, participants read an article, ostensibly from “*Genomic Scientech,*” indicating that humans share 99.9% of their genetic material. The text of this article was adapted from an actual article in *American Psychologist* (Ossorio and Duster, 2005). The article provided various forms of genomic evidence that genetic overlap between humans is high. In the low overlap condition, the article was kept as similar as possible, but certain words and numbers were modified to reflect the view that genetic overlap between humans with ancestry from different parts of the world is low (21.4%). The article suggested that race is a legitimate biogenetic construct and that races can be thought of as similar to extended families.

**FIGURE 4:**
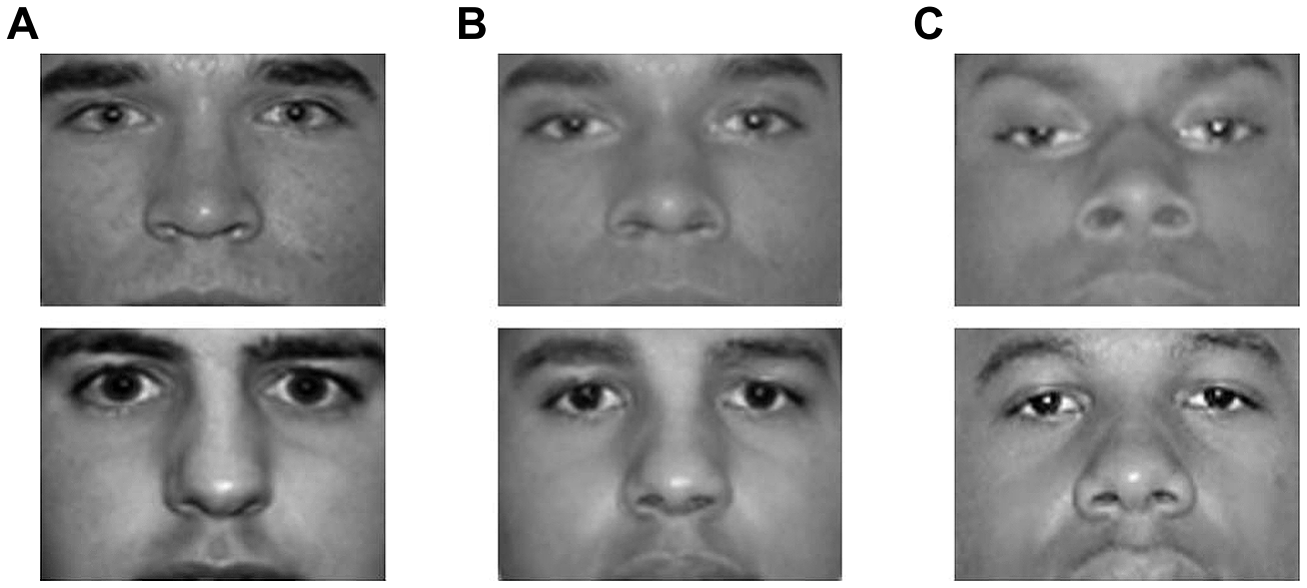
**Sample photographs from Study 3.** Stimuli used in this study included 3 photos of 100% White targets (column **A**), 3 digitally created photos of 50% Black/50% White targets (column **B**), and 3 photos of 100% Black targets (column **C**). Images were cropped to display only the targets’ eyes and noses.

Stimuli in the face judgment task were nine grayscale images of male targets. Six photos of 100% Black and 100% White targets (three of each race) were drawn from the same normed and standardized photo set used to create the videos used in Study 1 (Goff et al., 2008). Three additional photos of 50% Black/50% White targets were created by digitally combining three Black and White face pairs. Images used in this study were cropped to display only the targets’ eyes and noses; hair and mouths were not visible (see **Figure 4** for sample stimuli).

#### Procedure

After providing informed consent, participants began the first of what was described as two separate studies. “Study 1” was described as a reading comprehension task. The computer randomly presented one of two articles – high genetic overlap or low genetic overlap. After reading their assigned article, participants completed a manipulation check item: “In your estimation, if you drew two people at random from the entire world, what percentage of genetic material would they have in common?” Participants selected from five different values ranging from 1 (21.4%) to 5 (99.9%). In addition, participants were asked to describe the article in their own words.

Next, in “Study 2,” the computer presented the following instructions: “Research in psychology has begun to show that people are surprisingly good at making judgments about a person just by looking at their face. We are interested in the accuracy of people’s facial judgments. We will ask you to make judgments about different people just by looking at their face. In addition, we will ask you to estimate how confident you are about your rating.”

Next, participants saw a total of nine faces (three White, three Black, three Biracial), presented individually in random order. Participants were asked to rate each face on a randomized series of positive and negative traits using a 1–7 rating scale (1 = *not at all*…7 = *extremely*). The traits were: horrible, aggressive, dangerous, annoying, mean, crazy, wild, emotional, compassionate, polite, pleasant, nice, trustworthy, kind, calm, and rational. Participants also indicated how certain they were in their ratings (1 = *extremely uncertain*…5 = *extremely certain*).

Next, participants completed a battery of questionnaires, including demographics, political orientation (one item: -5 = *extremely conservative*…5 = *extremely liberal*), and a measure of the Big Five personality traits [the Big Five Aspects Scale (BFAS), DeYoung et al., 2007]. A suspicion check item asked participants to describe in their own words what they believed the study was about. Finally, participants were guided through a full debriefing which explained that the text read during “Study 1” was created for this experiment and not an actual, scientific report.

### Results

#### Suspicion and Manipulation Checks

##### Suspicion check

Two coders rated responses to the suspicion check item on whether participants guessed that the study concerned the relationship between beliefs about genetics and perceptions of race. The coders (who achieved 99% agreement) identified 19 participants who identified the correct purpose of the study. These participants wrote statements such as, “This study was about what people think about genetics and how it affects racism.” Because knowledge of the hypothesized relationship between genetic overlap belief and racial perception could contaminate participants’ responses, these participants were removed from the analyses.

##### Genetic belief manipulation check

Of the remaining participants, seven failed the genetic belief manipulation check by leaving the item blank and six failed by summarizing the article in a non-sensical fashion. This left a total of 139 participants, 62 in the high overlap condition and 77 in the low overlap condition. Among these participants, the manipulation appeared to be successful: high overlap participants’ estimates of the percentage of genetic material people have in common (*M* = 5.0, SD = 0.01, corresponding to a mean estimate of 99.9%) were significantly higher than those of low overlap participants (*M* = 1.13, SD = 0.62, corresponding to a mean estimate of 22%), *t*(1,137) = 49.55, *p* < 0.001.

#### Trait Evaluations

A factor analysis of the trait items revealed two significant factors (eigenvalues > 5.0). One factor – which included all items but “rational” – reflected overall valence (positive vs. negative traits). The second factor included only the traits “dangerous,” “crazy,” “emotional,” and “horrible.” These four traits (Cronbach’s α = 0.72), which appeared to cluster specifically around “primitive” or animal-like concepts, were combined into an evaluation index, our main dependent measure. To examine the effects of target race on participants’ evaluations, we conducted a 2 (genetic overlap belief: high vs. low) × 3 (face type: 100% White, 50% White-50% Black, 100% Black) analysis of variance, with repeated measures on the second factor. This analysis revealed a significant effect for face type, *F*(2,136) = 16.87, *p* < 0.001, $\eta_{p}^{2}$ = 0.20, indicating that participants did not rate the three faces equivalently. In addition, the predicted genetic overlap belief × face type interaction also emerged, *F*(2,136) = 4.52, *p* < 0.05, $\eta_{p}^{2}$ = 0_._06.

As reported in **Table 2**, participants in the low genetic overlap condition rated the biracial targets more negatively on the four-item “primitivism” index (*M* = 3.05, SD = 0.69) than did participants in the high overlap condition (*M* = 2.78, SD = 0.82), *F*(1,137) = 4.33, *p* < 05, *d* = 0.35. Low and high overlap participants did not differ in their ratings of 100% Black and 100% White targets (both *F*s < 1.0).

**Table 2:** Study 3 evaluations.

	100% White	50% White-50% Black	100% Black
High overlap	3.24 (0.86)	2.78 (0.82)	2.95 (0.82)
Low overlap	3.23 (0.80)	3.05 (0.69)	2.93 (0.91)

*Mean evaluation ratings (dangerous, crazy, emotional, horrible) of monoracial and biracial target persons as a function of genetic overlap belief condition in Study 3. SDs appear in parentheses.*

Participants in the high overlap condition rated the biracial target (*M* = 2.78, SD = 0.82) less negatively on the four-item index than the 100% Black target (*M* = 2.95, SD = 0.82), *t*(1,137) = 2.18, *p* < 0.05, *d* = 0.33. In contrast, participants in the low overlap condition rated the biracial target (*M* = 3.05, SD = 0.69) equivalently to the 100% Black target (*M* = 2.94, SD = 0.91),* t*(1,137) = 1.52, *p* = 0.13.

Additional analyses that used as the dependent variable an index of all traits which clustered around a positivity-negativity dimension did not yield a significant genetic overlap belief × face type interaction, *F*(2,136) = 1.73, *p* = 0.18. Thus, it appears that genetic overlap beliefs were better predictors of participants’ ratings on traits that generally seem more “biological” and thus more susceptible to genetic influence. Indeed, these types of traits (rather than generalized negativity) are precisely those that feature prominently in stereotypes about the assumed “primitive” nature of people of African descent (e.g., Goff et al., 2008).

Note that whereas in Study 1 low overlap participants displayed more neural signs of avoidance in response to biracial targets than to Black targets, in the present study, low overlap participants’ explicit ratings of the two types of targets were equivalently negative. This difference may be due to differences between the two paradigms (e.g., general approach/avoidance vs. specific trait ratings; implicit vs. explicit measures). However, the overall pattern is consistent with our primary hypothesis: low overlap participants rated biracial targets more negatively than did high overlap participants.

##### Political orientation

Recent results indicate that political conservatism predicts the tendency to perceive biracial faces as Black (Krosch et al., 2013). Might our results be explained by the fact that the low genetic overlap participants were more conservative? The data do not support such an explanation. First, we re-conducted the analysis with political orientation included as a covariate. In this analysis, the genetic overlap belief × face type interaction remained significant, *F*(2,139) = 3.50, *p* < 0.05. Second, the fact that genetic overlap beliefs were manipulated renders an explanation due to individual differences in political orientation implausible. Moreover, the belief × face type interaction remained significant when each of the Big Five traits were added as covariates.

##### The 100% White targets

As depicted in **Table 2**, both high and low genetic overlap participants rated the 100% White targets significantly more negatively than both the biracial and Black targets, all *F*s > 3.72, all *p*s < 0.05. Why might this be the case? We considered the possibility that participants may have judged the White faces as less attractive than the biracial or Black faces. However, a pilot study (*N* = 147) indicated no significant effect of face race on ratings of attractiveness, *F*(2,147) = 0.55, *p* = 0.58. Another possibility may have to do with self-presentational concerns. Future researchers may consider examining whether, for example, extrinsic motivation to respond without prejudice (Plant and Devine, 1998) predicts ratings of White targets relative to Black or Biracial targets.

Nonetheless, this pattern does not contradict our hypothesis that the effects of genetic overlap beliefs would be primarily evident with biracial targets. This was the case across all studies. Whereas in Study 1 the difference between low and high overlap participants in negativity toward biracial targets was expressed in autonomic neural reactions, in Study 3 this difference was expressed in explicit ratings of the target’s negative traits. Note that in Study 2, genetic overlap beliefs did not affect participants’ ratings of biracial targets’ attractiveness. Thus, low overlap participants’ avoidance response in Study 1 and negative trait ratings in Study 3 do not appear to be related to their perceptions of the targets’ physical attractiveness. Instead, based on Study 3’s data, it appears that low overlap participants are more likely to ascribe negative, underlying (invisible) personal qualities to biracial people.

### Discussion

These studies – drawing on both undergraduate and community samples – represent, to our knowledge, the first evidence that how laypeople mentally quantify the genetic aspect of race is an important predictor of approach and avoidance reactions to biracial targets. Whereas previous researchers have examined the effects of, for example, essentialist beliefs (Chao et al., 2013) and beliefs regarding nature vs. nurture (No et al., 2008; Williams and Eberhardt, 2008) on stereotyping-related processes, the present work goes an important step further by beginning to specify how people mentally quantify “nature,” and how this, in turn, relates to evaluative and approach/avoidance tendencies toward racially-ambiguous individuals. In Study 1, individuals who believed in lower (vs. higher) genetic overlap showed higher levels of neural avoidance from biracial individuals. In Study 2, individuals who believed in lower (vs. higher) genetic overlap took longer to classify biracial (vs. monoracial) targets, suggesting that they experienced greater difficulty. In Study 3, low overlap participants explicitly rated biracial targets more negatively than did high overlap participants. In addition, the Study 3 finding that randomly manipulated genetic overlap beliefs yielded results that were in line with the continuous, individual difference variable suggests that these beliefs play an important role in *causing* different patterns of evaluation of biracial targets. As such, these findings shed light on a unique source of bias against biracial people, who tend to be racially-ambiguous, and constitute one of the fastest growing minority groups in the United States (Townsend et al., 2009).

These data suggest that a mental model of high genetic distinctiveness creates an expectancy of low racial ambiguity. That is, if individuals are clearly distinct from one another at the genetic level, they should be easy to categorize. An ambiguous person violates this expectancy, which, in turn, appears to elicit a negative response.

In both Study 1 and Study 2, we found that a belief in low genetic overlap predicted parallel results, regardless of whether the question was about people drawn at random from the whole world or people from the same race. It was conceivable *a priori* that the two genetic overlap questions (general vs. within-group) would be inversely correlated (i.e., the more similar group members are to each other, the more different they are from members of other groups). The data clearly indicated otherwise: the two genetic overlap questions were highly, positively correlated, and both yielded similar results. Thus, it appears that people with a low within-group overlap perspective expect even members of the same group to possess considerable genetic difference. The greater the assumed difference, the greater the avoidance of a face that blurs the distinction between the race of one person and the next. Taking both estimates together, the Study 1 data suggest that a generalized expectation of low genetic overlap leads perceivers to view ambiguous or difficult-to-classify individuals negatively.

One limitation of the current studies is that our experimental designs and participant samples make it impossible for us to examine the effects of participant race on genetic overlap estimates and perceptions. When we asked participants to provide genetic overlap estimates (Studies 1 and 2), or when we manipulated them in a general way without naming a specific group (Study 3), we have no way of knowing what groups participants have in mind when making judgments. In future research it would be helpful to examine variability in estimates depending on which racial groups participants are asked to think about. Participants may, for example, estimate higher overlap between their racial group and some out-groups, but lower overlap between their racial group and others. These estimates may vary based on the relative status of the out-group in question, or perhaps even on group-esteem. For example, individuals with high racial-group-esteem may perceive more overlap with high-status groups, whereas those with low group-esteem may perceive less overlap with high-status groups. Even when considering only racial out-groups, people likely perceive some groups as more similar and others as less similar (e.g., perhaps based on similar phenotypic features or geographic proximity). All of this variation may have important implications for attitudes about and interaction with members of racial in- and out-groups. Further, future research which specifically recruits large and equal samples of specific racial groups could shed more light on whether people draw on genetic overlap estimates differently when thinking about members of their own racial groups vs. members of racial out-groups.

Throughout this paper, we have argued that a belief in low genetic overlap is associated with a variety of negative reactions to biracial individuals. In future research it will be important to investigate more systematically how differences in scientific literacy or even general IQ factor into how people respond to questions about genetic overlap. People who respond with lower estimates are more out of step with the scientific data, suggesting lower scientific or genetic literacy. More generally, scholars have long posited a negative relationship between cognitive ability and prejudice (e.g., Adorno et al., 1950; Allport, 1954; Kutner and Gordon, 1964; Hodson and Busseri, 2012), and there has been a recent call for further examination of this relationship and its possible underlying mechanisms (Dhont and Hodson, 2014; Hodson, 2014).

In addition, future researchers should investigate which exact emotions are associated with the negative responses suggested by these studies (e.g., fear, disgust), as well as examine the impact of other types of genetic beliefs such as, for example, the amount of genetic overlap between men and women, normal weight and obese individuals, or individuals from different socioeconomic backgrounds. Finally, future studies should also capitalize on the finding in Study 3 that genetic beliefs can be manipulated as an avenue for prejudice-reduction interventions (see also Plaks et al., 2012, Study 2). Teaching basic principles of human genetics could prove to be a simple, inexpensive, and powerful way to reduce racial bias.

## Conflict of Interest Statement

The authors declare that the research was conducted in the absence of any commercial or financial relationships that could be construed as a potential conflict of interest.

## Footnotes

1 In other studies (including Study 2 below), we have included a question that asks specifically for an estimate of genetic variation between members of two racial groups. We have found, however, that this item yields inconclusive results, likely due to social desirability concerns raised by asking participants to make direct between-race comparisons. However, we consider the “entire world” question a plausible proxy for estimates of between-race genetic overlap. Most likely when people sample two individuals from the entire planet, they generally select people from distant locations who are of different races.

2 Conducting this same analysis with political orientation added as a covariate yielded a similar pattern of results. Controlling for political orientation in this analysis yielded a significant interaction between time and estimates of genetic overlap shared within racial groups (question “2”: *F*(2,42) = 4.07, *p* < 0.05, $\eta_{p}^{2}$ = 0.16), but weakened the interaction between time and estimates of genetic overlap shared between racial groups (Question “1”: *F*(2,42) = 2.59, *p*= 0.09, $\eta_{p}^{2}$ = 0.11. The quadratic trends for each of the interactions between time and genetic overlap estimates remained significant: general overlap estimate Question “1,” *F*(1,21) = 4.84, *p* < 0.05, $\eta_{p}^{2}$ = 0.19; within-race overlap estimate question “2,” *F*(1,21) = 7.55, *p*= 0.01, $\eta_{p}^{2}$ = 0.26. No significant effect of political orientation was observed, *F*< 1.

3 Additional analyses that included Question 3 (estimates of genetic overlap between two people explicitly identified as belonging to different races) did not yield any significant effects and will not be discussed further.

4 Including participants’ score on the genetics quiz as a covariate did not alter the results. Thus, this variable will not be discussed further.

5 The absence of a face type × classification condition interaction, *F*(1,386) = 0.17, *p* > 0.65, represents a failure to replicate Halberstadt and Winkielman’s (2014; Study 3) finding. We would note that the present study had 6.34 times as many participants (*N* = 393) as Halberstadt and Winkielman’s (*N*= 63).
